# Preventing Crohn’s recurrence, Kono-S and extended mesenteric excision: network meta-analysis category

**DOI:** 10.1007/s00423-026-04103-5

**Published:** 2026-07-15

**Authors:** Talia Shepherd, Devansh Shah, Joseph Do Woong Choi, Toufic El-Khoury, Nimalan Pathma-Nathan, Amy Cao, James Wei Tatt Toh

**Affiliations:** 1 Department of Colorectal Surgery, Westmead Hospital, Westmead, NSW Australia; 2 The University of Sydney, Nepean Hospital, Penrith, NSW Australia; 3 The University of Sydney, Westmead Hospital, Cnr Mons and Darcy Rd, NSW 2145 Westmead, Australia; 4 The School of Medicine, University of Notre Dame, Sydney, NSW Australia

**Keywords:** Kono-S, Crohn’s, Mesentery, Anastomosis, Recurrence

## Abstract

Introduction

Crohn’s disease (CD) is a complex disease with rising incidence that is usually treated with medical management. However, surgery plays an important role for medically refractive cases, or Crohn’s related complications. New techniques such as extended mesenteric excision (EME), and Kono-S anastomosis (KSA) are being increasingly adopted. However, their potential for reducing CD recurrence remains unclear.

Method

Studies were identified through searching Medline, Embase, Cochrane Library and JBI EBP from database inception to January 2025. Studies where patients with CD had intestinal resection and underwent at least two of four interventions being KSA, other anastomosis, EME and/or limited mesenteric excision (LME) were included. Two investigators independently identified studies and abstracted data. RCT quality was assessed using Cochrane Risk of Bias 2 tool and cohort study quality assessed using Newcastle Ottawa Scale. The study adhered to PRISMA guidelines. Bayesian network meta-analysis was conducted to assess risk of surgical and endoscopic recurrence at 5 years and 6–12 months respectively.

Results

Thirteen studies composed of 3 RCTs and 10 cohort studies (*n* = 2,044) were included, assessing surgical and/or endoscopic recurrence after KSA, other anastomosis, EME or LME. There was statistically significant reduction in surgical recurrence in KSA compared to other anastomosis (odds ratio 0.07, 95% credible interval) but not EME compared to LME. There was no difference in endoscopic recurrence in any groups.

Conclusion

KSA was associated with reduced surgical recurrence compared with other anastomosis, but not endoscopic recurrence. There were insufficient studies to support similar improvement in recurrence in EME over LME.

**Supplementary Information:**

The online version contains supplementary material available at https://doi.org/10.1007/s00423-026-04103-5.

## Introduction

Crohn’s disease (CD) is a condition characterised by lesions predominantly along the gastrointestinal tract. It has an incidence of 3–20 per 100,000 patients where the incidence has plateaued in western countries, but is rising in developing nations [1]. It has a multifactorial pathogenesis with attributions to the autoimmune, genetic, environmental (including the gut microbiome), and gut-immune axis factors [2].

Traditionally, CD is initially managed by medical therapy with surgery reserved for refractory cases, or due to complications such as fibrotic strictures, intrabdominal abscesses, fistulae, perforation and malignancy. Despite advances in medical management with biologic therapy, disease recurrence rates are high with up to 86% at 10 years and one third of these patients’ requiring surgery [3]. However surgery does not prevent recurrence with 17.7% and 31.3% of patients at 5 and 10 years respectively requiring a repeat resection [4] Recently the treatment paradigm for uncomplicated ileo-colic disease has shifted back towards a surgery-first approach as outcomes from minimally invasive techniques improve, with the revised European Crohn’s and Colitis Organisation (ECCO) guidelines suggesting a laparoscopic resection is a suitable alternative to infliximab or adalimumab in limited terminal ileal or ileocolic non-stricturing CD [5].

As our understanding of the disease pathophysiology changes, there has been interest in developing new surgical techniques to reduce disease recurrence, prevent repeat resection, and improve quality of life.

The standard anastomotic configuration is the stapled side to side anastomosis (STSA) [5] however in 2003, Dr Toru Kono first performed the Kono-S anastomosis (KSA), with its outcomes first published in 2011 [6]. It involved a handsewn 7 cm wide, antimesenteric transverse anastomosis which excluded the mesentery from the anastomosis with supporting columns that prevented mesenteric distortion and close mesenteric mobilisation to preserve the mesentery and neovascular supply. The KSA operated on the principle of excluding the mesentery from the anastomosis based on the observation that CD recurrence occurred on the mesenteric border [7].

Whether the mesentery plays a critical role in driving the disease process is contentious since Coffey et al. published their cohort study describing an extended resection of the mesentery [8]. The mesentery contains neurovasculature, lymphatics, stromal cells, and fibroblasts which secrete adipokines and neuropeptides, hence potentially contributing to immunomodulation and chronic inflammation.[9, 10] By resecting the affected mesentery at the root, they demonstrated a significant reduction in surgical disease recurrence compared to a standard STSA.

The efficacy of these techniques remains controversial and it is difficult for the surgeon to decide which to adopt if any, when operating on Crohn’s patients. Subsequent larger cohort studies and randomised controlled trials have published results both for and against each method with regards to disease recurrence. Unfortunately, most studies are only two-arm comparing either type of anastomosis (KSA vs. other) or approach to the mesentery (LME vs. EME) but few have compared these approaches to each other. The aim of this study was to compare the relative effectiveness of all four surgical techniques regarding disease recurrence.

## Methods

**Fig. 1:**
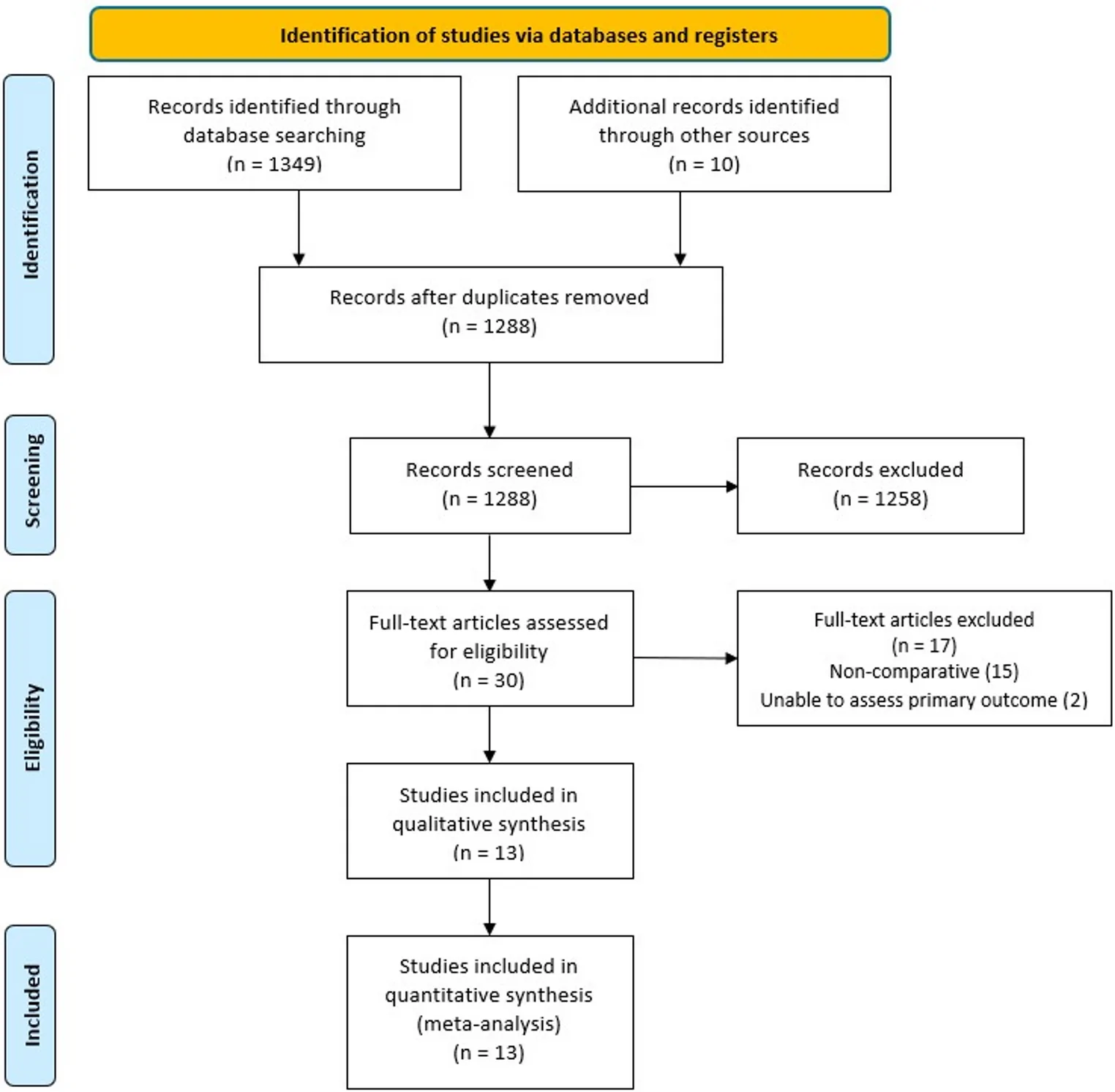
PRISMA Flow diagram [11]

The network meta-analysis was performed according to the Preferred Reporting Items for Systematic Reviews and Meta-Analyses (PRISMA) guidelines (Fig. 1). [11] The study was registered with PROSPERO (International Prospective Register of Systematic Reviews, registration number CRD42024499493).

### Literature search

We conducted a systematic literature search using Embase, MEDLINE, the Cochrane Central Register of Controlled Trials, and the Joanna Briggs Institute Evidence Based Practice (JBI EBP) Database from inception to January 2025. Only adult human studies reported in English were included. Participants were patients who underwent an intestinal resection for CD. Studies must have included a comparison of at least two of the four interventions (KSA, other anastomosis, EME or LME) following the PICO scheme. We used a combination of subject words and free words for our search strategy which included (1) Crohn’s disease” OR Crohn* AND (2) Kono-S or Kono* OR (3) mesenteric resection or mesenter* resect* or mesenter* excis* or mesenter* remov* or mesenter* cons*.

### Study selection

After excluding duplicates in EndNote Clarivate, primary studies’ titles and abstracts underwent independent blinded assessment by two authors (TS and JC) using Rayyan software. Recent high yield conference abstracts were screened given the rapidly evolving evidence in this field. The inclusion criteria consisted of the following: (1) studies evaluating patients who had undergone intestinal resection due to CD; (2) studies where at least two of the interventions measured were compared including KSA, standard anastomosis (as defined by the authors and including end to end, end to side and STSA), extended mesenteric excision (EME) or limited mesenteric excision (LME) (3) randomised clinical trials (RCTs) and cohort studies, and (4) studies where CD recurrence was reported. Review articles, case reports, cohort studies with a single arm, and studies where disease recurrence was not specifically described were excluded. Discrepancies between the two reviewers were resolved by consensus from the senior author (JT).

### Data extraction

We extracted data on the baseline patient characteristics, study exclusion criteria, operative details including type of anastomosis or approach to the mesentery and post-operative details including disease recurrence rate and method of detecting disease recurrence, follow-up time and overall complications.

### Risk of bias

The risk of bias was independently assessed by two authors (TS, DS) with RCTs assessed using the Cochrane Risk of Bias 2 Tool (RoB 2), and cohort studies gauged with the Newcastle-Ottawa Scale (NOS). [11, 12]. Any disagreements were resolved by discussion and consensus between all authors.

### Outcome assessment

The primary outcome measure was the incidence of surgical recurrence at 5 years. The secondary outcome was endoscopic recurrence at 6–12 months. Clinical recurrence could not be assessed due to insufficient data.

### Statistical analysis

A network meta-analysis was performed using a Bayesian Markov chain Monte Carlo method in WinBUGS 1.4.3 (MRC Biostatistics Unit, Cambridge, United Kingdom) through the conduit of the Microsoft Excel-based macro NetMetaXL 1.6.1 (Canadian Agency for Drugs and Technologies in Health). A convergence test was conducted for each analysis by checking whether the Monte Carlo error was < 5% of the standard deviation (SD) of the effect estimates or the variance between the studies. Convergence was achieved for all analyses at 20,000 “burn in” runs and 30,000 model runs.

Surgical and endoscopic recurrence rates were calculated by the pooled estimates of odds ratios (OR) and 95% confidence intervals (CIs) of direct comparisons between KSA, other anastomosis, EME and LME. Both direct and indirect evidence for all intervention groups’ comparisons were combined to estimate the examined outcomes with a 95% equal-tail credible interval (CrI). To represent the results visually, representation of probabilities of network plots with network nodes and lines, and forest plots were generated by the gemtc package in STATA (Stata MP, version 15; StataCorp LP). Pairwise comparison tables and graphical rankograms were generated using NetMetaXL. Along with rankograms, the relative efficacy of different intervention groups for preventing CD recurrence (surgical and endoscopic) was presented with their surface under the cumulative ranking (SUCRA). SUCRA values ranged from 0 to 1, with 0 having the highest chance of being ranked lowest and 1 having the highest chance of being ranked best. All results have been presented as relative effects (OR) and Bayesian estimates of the probability of developing CD recurrence.

## Results

**Fig. 2:**
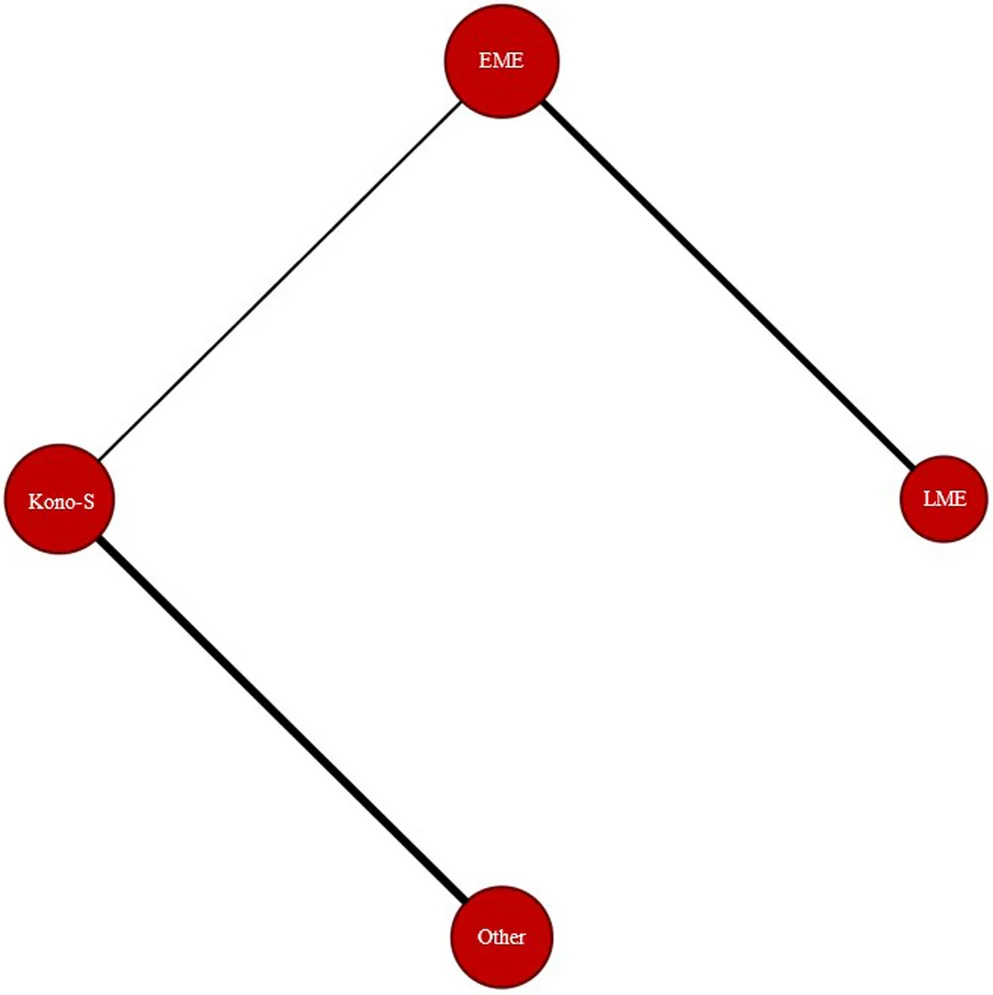
Network plot for available direct comparisons and network of trials for primary outcome of surgical recurrence after KSA, other anastomosis, EME, and LME

**Fig. 3:**
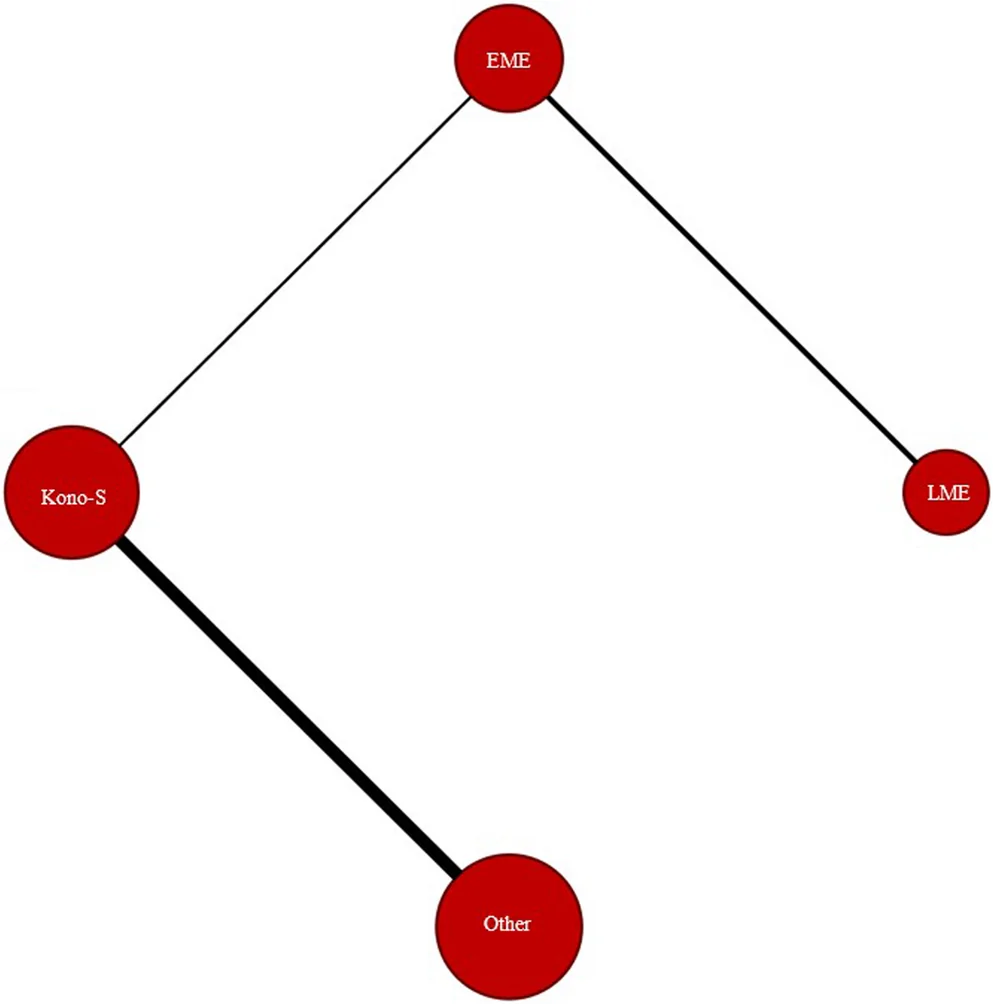
Network plot for available direct comparisons and network of trials for secondary outcome of endoscopic recurrence after KSA, other anastomosis, EME, and LME

The search strategy identified 1288 studies (excluding duplicates) from four databases. After applying inclusion and exclusion criteria, 13 studies (3 RCTs and 10 cohort studies) were included (see PRISMA flow diagram – Fig. 1).^11^ Network plots of the 8 studies for the primary outcome of CD surgical recurrence incidence and 10 studies for the secondary outcome of CD endoscopic recurrence are shown in Figs. 2 and 3 respectively.

Of the included studies, one RCT (Trencheva et al.) and one cohort study (Uchino et al.) had preliminary results published in abstract format from the 2024 ECCO conference [13, 14].

Table 1 describes the characteristics of the 13 studies included in analysis (see full table in Supplementary Table S1). These 13 studies dated from 2011 to 2024 and included 1217 participants in the surgical recurrence group and 1502 participants in the endoscopic recurrence group, ranging from 51 to 326.

**Table 1:** Study characteristics of studies includes in network meta-analysis

Study	Country	Published evidence	Comparators	Design	Data collection time period	Population	Intervention	Primary Outcome
Kelm et al. 2022 [26]	Germany	Full journal article	KSA vs. STS (stapled)	Retrospective cohort study	2019–2021	51 patients Age 37 (16–71) M: F 28:23 Mean BMI 23.45	Ileocolic resection. KSA (*n* = 22) vs. STS (*n* = 29).	No significant difference in ER (STS 13 vs. KSA 7; *p* = 0.36)
Shimada et al. 2019 [23]	Japan	Full journal article	KSA vs. ETEA	Retrospective cohort study	2006–2016	215 patients Age 37 (30–45) M: F 158:57 BMI 18.7 (16.9–20.2)	KSA (*n* = 117) vs. handsewn ETEA (*n* = 98)	SR significantly lower in KSA (4 vs. 24, *p* < 0.001)
Kono et al. 2011 [26]	Japan	Full journal article	KSA vs. other	Retrospective cohort study	1993–2003	142 patients Age 30 (14–62) M: F 115:27	KSA (*n* = 69) vs. other (*n* = 73) (handsewn ETEA or STS or stapled STS) with or without stricturoplasty	No significant difference in ER at 1 year (KSA 49/59 vs. other 58/73, *p* > 0.05) or 5 years (KSA 69/69 vs. other 73/73). Significantly lower SR in the KSA group at 5 years (0 vs. 11; *p* = 0.0013).
Coffey et al.Cpffrecccc 2018 [8]	Ireland	Full journal article	EME vs. LME	Retrospective cohort study	2004–2010 (LME), 2010–2012 (EME)	64 patients Age 36 (24–51) M: F 28:36	EME (*n* = 34) vs. LME (*n* = 30).	SR significantly lower in EME group (1 vs. 9, *p* = 0.003)
Mineccia et al. (REMEDY) 2022 [27]	Italy	Full journal article	EME vs. LME	Retrospective cohort study	2009–2019	326 patients Age 40.6 (24.6–56.6)	EME (*n* = 204) vs. LME (*n* = 122).	No difference in ER (EME 91 vs. LME 57, *p* = 0.7) or USS recurrence (EME 40.4% vs. 41.1%, *p* = 0.9). No difference in SR (EME 2.8% vs. 4%) at 5 years.
Luglio et al. (SUPREME-CD) 2020 [18]	Italy	Full journal article + abstract of the trial update (ECCO conference)	KSA vs. STS (stapled)	single blind RCT (only patients blinded)	2015–2017	119 patients Age 38.9 (25–60)	KSA (*n* = 51) vs. STS (*n* = 68).	Significantly lower ER at 6 months in KSA group (17 vs. 51, *p* < 0.001). Significantly lower SR at 54 (42–60) months in KSA (0% vs. 8.8%, *p* = 0.02)
Zhu et al 2021 [28]	China	Full journal article	EME vs. LME	Retrospective cohort study	2000–2018	126 patients Age 30.77 (SD 10.21) BMI 18.2 (SD 2.76)	EME (*n* = 66) and LME (*N* = 60).	Significantly lower SR in EME group (7 vs. 18, *p* = 0.01). This differencestayed the same when subgroup analysis was done on only patients who had an anastomosis (this study did not exclude stomas) (EME 6 vs. LME 14, *p* = 0.01).
Alibert et al. (KOCORICCO) 2023 2p. D [22]	France	Full journal article	KSA vs. STS	Multicentre prospective, propensity matched cohort study- retrospective STS cohort vs. prospective KSA	2020–2022 (prospective) and 2013–2015 (retrospective)	183 patients Age 35 (27–50) M: F 81:102 BMI 21.2 (19–24.9)	KSA (*n* = 61) vs. STS (*n* = 122)	No difference in ER (KSA 29 vs. STS 54, *p* = 0.67)
Holubar et al. 2024 [5, 16. 17]	USA	Full article and abstract P420 from ECCO conference 2023	KSA vs. EME vs. KSA + EME	Retrospective cohort study	2015–2022	186 patients Age 37.5 (± 15.3) M: F 90:96 BMI 25.5 ± 5.8	KSA (*n* = 74) vs. EME (*n* = 66) vs. KSA + EME (*n* = 46).	No difference in ER (KSA 4/20 vs. EME 6/31 vs. KSA + EME 3/18, *p* = 0.69) or SR (KSA 1 vs. EME 0 vs. KSA + EME 1, *p* = 0.22)
Van Der Does De Willebois et al. (SPICY trial) 2024 [21]	Netherlands and Italy	Full journal article	EME vs. LME	RCT double blind (patient and endoscopic scoring), multicentre	2020–2023	131 patients Age 36 (IQR 25–54)	EME (*n* = 66) vs. LME (*n* = 65).	No difference in ER (EME 28 vs. LME 28, *p* = 1)
Trencheva et al. 2024 [13]	International multicentre (USA, Germany, Italy)	Abstract OP20 from ECCO conference 2024	KSA vs. STS	RCT	Not available	288 patients Age 34 (18–81) M: F 142:146 BMI 23.4 (SD 4.5)	KSA (*n* = 154) vs. STS (*n* = 134)	No difference in ER at 3–6 months (KSA 35/135 vs. STS 32/115, *p* = 0.883).
Tyrode et al. 2024 [20]	France	Full journal article	KSA vs. STS (all handsewn except for 1)	Combined cohort study - Kono S prospectively done, retrospective comparison cohort	2020–2022 (retrospective 2010–2020)	85 patients Age 34.9 (27.6–45.6) M: F 37:48 BMI 22.8 (IQR 19.9–26.2)	KSA (*n* = 30) vs. STS (*n* = 55)	No significant difference in ER using either Rutgeert’s score (KSA 17 vs. STS 27; *p* = 0.5) or modified Rutgeerts score (≥ i2b) (KSA 14 vs. STS 22, *p* = 0.55). No surgical recurrence in either group at 12 months.
Uchino et al. 2024 [14]	USA	Abstract P575 from ECCO conference 2024	KSA vs. STS (stapled)	Retrospective cohort study	2020–2022	174 patients BMI lower in the KSA group (24.3 vs. 26.4, *p* = 0.04)	KSA (*n* = 83) vs. STS (*n* = 91)	No difference in recurrence free survival between KSA and STS but multivariate analysis shows KSA associated with lower risk of perianastomotic recurrence requiring surgical/endoscopic intervention (OR 0.09, 95% CI 0.01–0.95, *p* = 0.05). ER not significant (KSA 12 vs. STS 14; *p* = 0.81).

Clinical recurrence data is included in Supplementary Table S2.

### Quality assessment

**Fig. 4:**
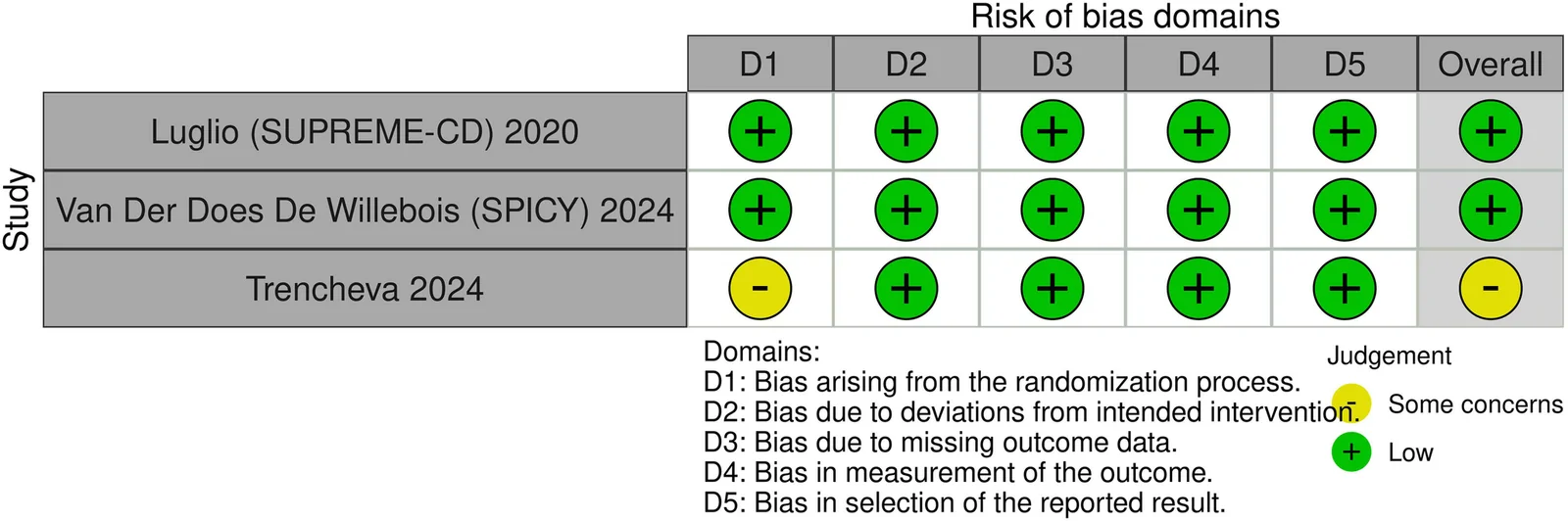
Risk of bias traffic light plot using robvis [15]

Cochrane RoB 2 found that two of the three RCTs were of a low risk of bias. The Trencheva et al. study was published as an abstract only and did not comment on the allocation sequence randomisation and concealment and there were some concerns in domain 1, leading to an overall quality rating of “some concerns” [13]. This is represented in Fig. 4 created using the Robvis tool [15]. The results of the NOS for cohort studies are summarised in Table 2. Eight of ten studies were of high quality, with the remaining two studies of moderate quality (awarded 6 points each). These two studies were the 2019 Shimada et al. study due to issues with comparability (difference in age and higher pre-operative biological therapy use in KSA group) and no comment on follow-up. The 2024 Uchino et al. had issues with comparability (higher post-operative biological therapy in KSA group) and no comment on length or loss to follow-up.

**Table 2:** Newcastle-Ottawa Scale for Cohort Studies. Each arrow represents a point awarded in the category

		Cohort Studies - Selection					Comparability		Cohort Study - Outcome			TOTAL SCORES
Author	Year of Publication	Representativeness	Selection of non exposed cohort	Ascertaining exposure	Outcome not present at start	Primary study control (age)		Additional factors (pre/post-op biologics)	Assessment of outcome	Long enough follow-up	Adequacy of follow-up	
Kelm et al. [26]	2022	↔	↔	↔	↔	↔		↔	↔	↔	-	8
Shimada et al. [23]	2019	↔	↔	↔	↔	-		-	↔	↔	-	6
Kono et al. [6]	2011	↔	↔	↔	↔	↔		-	↔	↔	↔	8
Coffey et al. [8]	2018	↔	↔	↔	↔	↔		-	↔	↔	-	7
Mineccia et al. REMEDY [27]	2022	↔	↔	↔	↔	↔		↔	↔	↔	-	8
Zhu et al. [28]	2021	↔	↔	↔	↔	↔		↔	↔	↔	-	8
Alibert et al. KoCoRICCO [22]	2024	↔	↔	↔	↔	↔		-	↔	↔	↔	8
Holubar et al. [16, 17]	2024	↔	↔	↔	↔	↔		↔	↔	-	-	7
Tyrode et al. [20]	2024	↔	↔	↔	↔	↔		↔	↔	↔	↔	9
Uchino et al. [14]	2024	↔	↔	↔	↔	↔		-	↔	-	-	6

### Surgical recurrence at 5 years

**Fig. 5:**
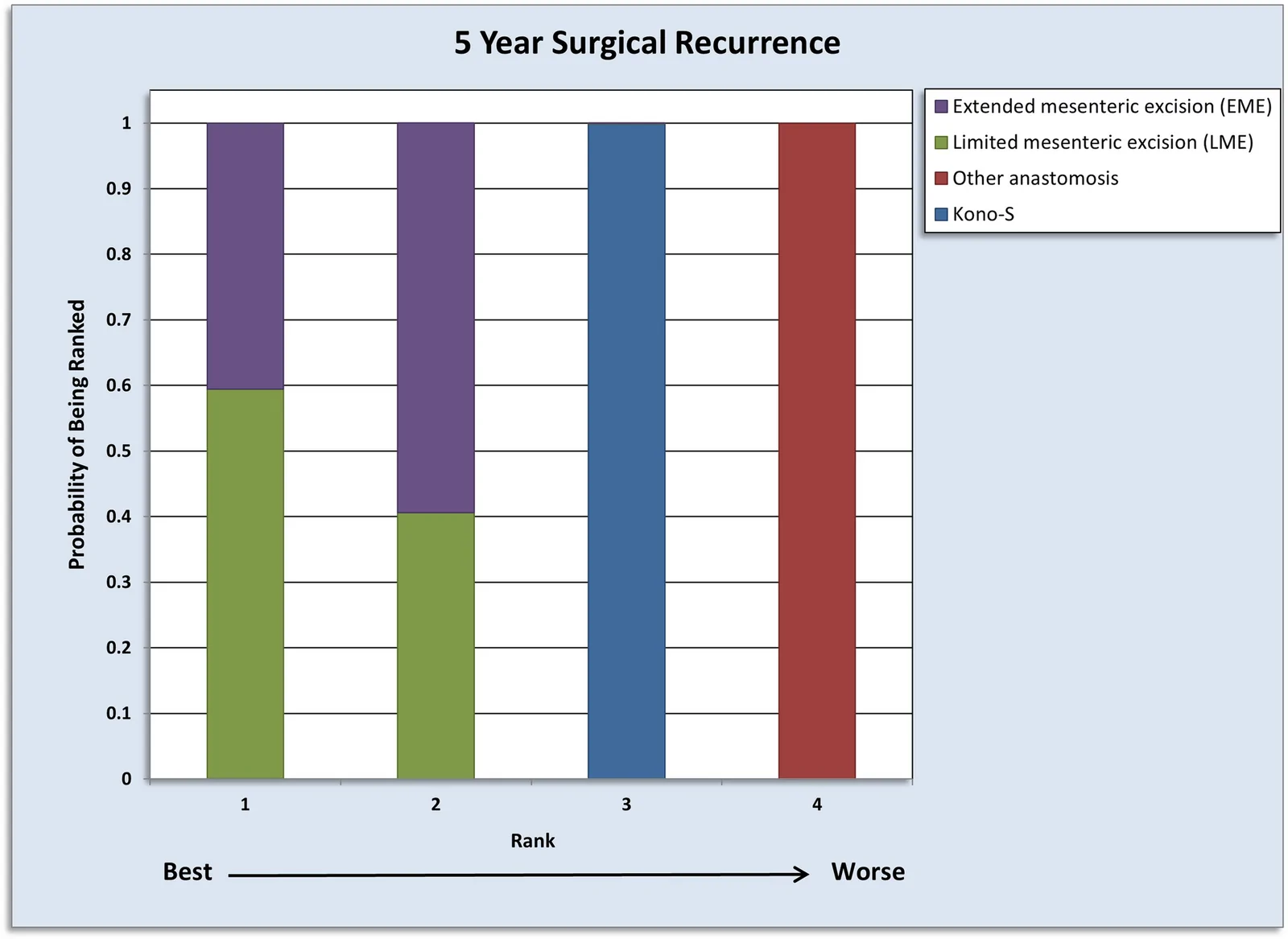
Rankogram with probability for ranking 1st to 4th (best to worst respectively) with regards to efficacy of KSA, other anastomosis, EME, and LME for reduction in CD surgical recurrence, ranging from 1.0 (most likely to fit in assigned ranking) to 0.0 (least likely to fit in assigned ranking) on the Y-axis. Ranking of interventions displayed on the X-axis ranging from 1st to 4th

**Fig. 6:**
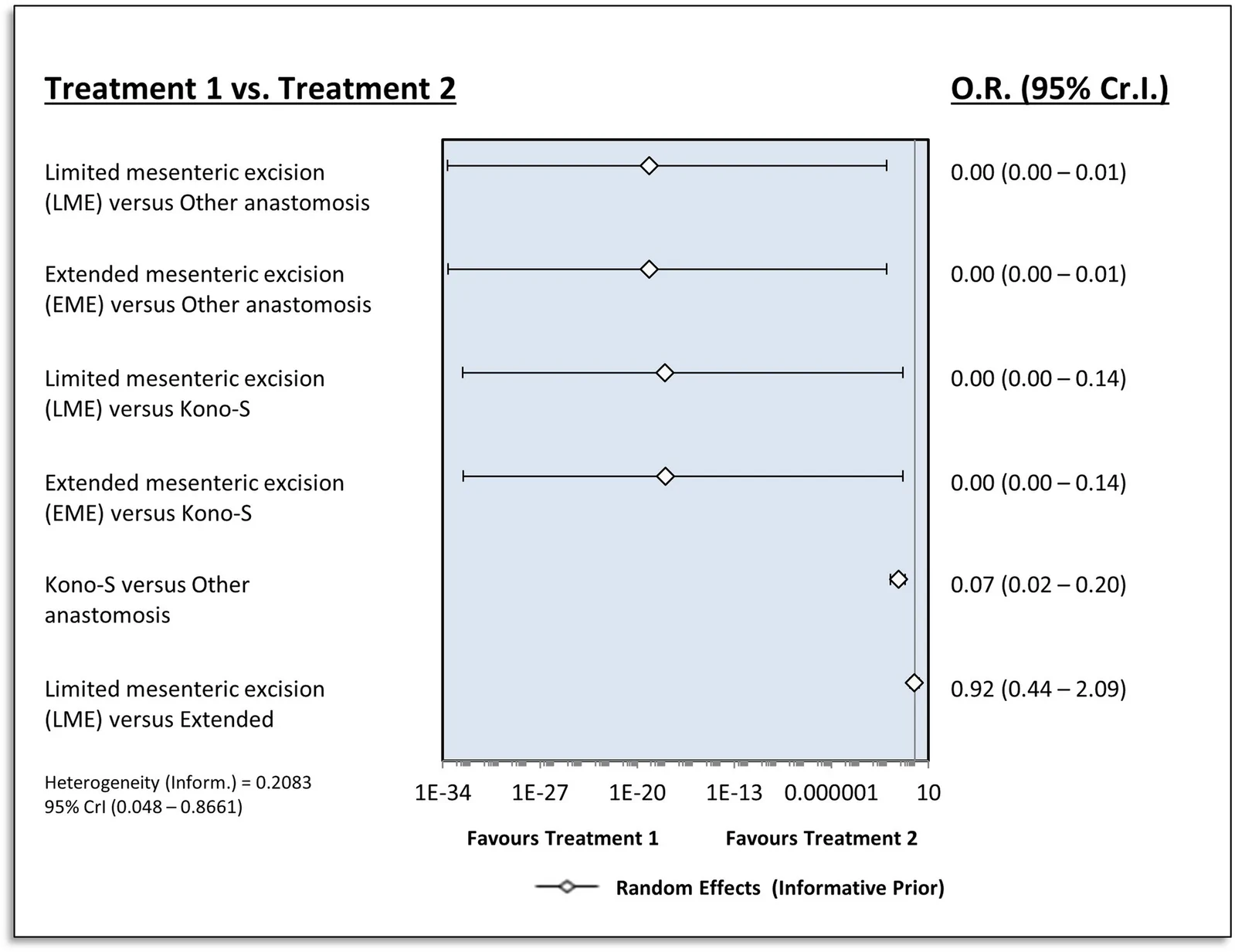
Forest Plot comparing interventions (KSA, other anastomosis, EME, and LME) assessed with relation to CD surgical recurrence. Line of no effect at OR = 1.0

There was a statistically significant reduction in the risk of surgical CD recurrence in the KSA group compared to other anastomosis (OR 0.07, 95% CrI 0.02–0.20). However, there was no significant difference in surgical recurrence in LME compared to EME (OR 0.92, 95% CrI 0.44–2.09). Although the network meta-analysis demonstrated a significant reduction in surgical recurrence in EME vs. KSA (OR 0.00, 95% CrI 0.00-0.14), LME vs. KSA (OR 0.00, 95% CrI 0.00-0.14), LME vs. other anastomosis (OR 0.00, 95% CrI 0.00-0.01), and EME vs. other anastomosis (OR 0.00, 95% CrI 0.00-0.01), this may not be clinically applicable. Only one study (Holubar et al. 2024) compared KSA to EME and only one recurrence event was reported in the KSA group, with none in the EME group, likely skewing the indirect comparisons in favour of EME and LME [16, 17] As such LME was ranked first with SUCRA 0.86, EME second (SUCRA 0.80), KSA third (SUCRA 0.33), and other anastomosis last (SUCRA 0.74 × 10^− 5^) as demonstrated in Table 3 and rankogram displayed in Fig. 5. Forest plot of intervention arm comparisons are shown in Fig. 6.

**Table 3:** SUCRA probabilities for efficacy of KSA, other anastomosis, EME, and LME for reducing CD surgical recurrence at 5 years

Treatment	SUCRA
Limited mesenteric excision (LME)	0.8644
Extended mesenteric excision (EME)	0.8017
Kono-S	0.3338
Other anastomosis	0.741 × 10^− 5^

Study characteristics of trials used for network meta-analysis and a pairwise comparison can be found in Supplementary Tables S3-S6, and Table 4 respectively.

**Table 4:** Pairwise comparison table – efficacy of KSA, other anastomosis, EME, and LME for reducing CD surgical recurrence at 5 years in network meta-analysis reported as odds ratio (95% credible interval). **Direct comparisons** are represented in bold, and *indirect comparisons* are represented in italics

Limited mesenteric excision (LME)			
0.92(0.44–2.09)	Extended mesenteric excision (EME)		
*0.00* *(0.00–0.14)*	0.00 (0.00–0.14)	Kono-S	
*0.00* *(0.00–0.01)*	*0.00* *(0.00–0.01)*	0.07 (0.02–0.20)	Other anastomosis

### Endoscopic recurrence at 6–12 months

There was no statistically significant reduction in the risk of endoscopic CD recurrence in the KSA group compared to other anastomosis (OR 0.78, 95% CrI 0.54–1.13). Similarly, there was no significant difference in endoscopic recurrence in EME compared to LME (OR 0.94, 95% CrI 0.53–1.68). There was also no significant reduction in endoscopic recurrence in KSA vs. EME (OR 1.06, 95% CrI 0.20–5.14), KSA vs. LME (OR 0.99, 95% CrI 0.17–5.42), LME vs. other anastomosis (OR 0.79, 95% CrI 0.14–4.72), and EME vs. other anastomosis (OR 0.74, 95% CrI 0.14–4.02). Whilst only one study (Holubar et al. 2024) compared KSA to EME, there were more endoscopic recurrence events was in both the KSA (4/20) and EME (6/31) groups, providing more reliable indirect comparisons [16, 17]. By SUCRA values, KSA was ranked first (0.63), EME was ranked second (0.59), LME ranked third (0.50), and other anastomosis ranked last (0.28) which is shown in Table S7 and rankogram displayed in Figure S1. Forest plot of intervention arm comparisons can be found in Figure S2.

Study characteristics of trials used for network meta-analysis and a pairwise comparison can be found in Supplementary Tables S8-S11, and Table S12 respectively.

## Discussion

This is the first Bayesian network analysis to simultaneously compare KSA, other anastomosis, EME and LME regarding their role in preventing recurrence of Crohn’s disease.

Although the KSA preserves the mesentery as much as possible, the anastomosis itself is on the antimesenteric side of the bowel, thus “excluding” it from the anastomosis. By virtue of anastomotic configuration, EME assumes the same principle by resecting the mesentery adjacent to the anastomosis.

At present, the 2024 ECCO guidelines recommend STSA, but suggested that the KSA can be an alternative approach after ileocolic resection [5]. This is based on the Oxford Centre for Evidence-Based Medicine Grade 3 evidence. However, they suggest that EME is not yet supported by evidence, based on Grade 4 evidence [5].

Our study found a significant reduction in CD surgical recurrence at 5 years in KSA vs. other anastomosis and ranked superior by SUCRA values 0.33 and 0.74 × 10^− 5^ respectively. Although no significant difference was found between LME and EME, both interventions were found by network meta-analysis to be superior to KSA and other anastomosis based on OR and SUCRA values. However, these findings need to be interpreted with caution. There were four studies directly comparing KSA to other anastomosis, three directly comparing LME to EME, one study comparing KSA to EME, and no study compared LME with KSA/other anastomosis, nor EME with other anastomosis. In addition, there was only one recurrence event in the KSA group and zero in the EME group and this one study formed the basis of indirect comparisons to generate OR and SUCRA values comparing KSA to LME, and other anastomosis with EME/LME, probably skewing results in favour of EME/LME and resulting in OR of 0.00. More studies directly comparing KSA with EME, LME and other anastomotic techniques would improve the statistical power to determine the optimal intervention for minimising surgical recurrence. Regarding endoscopic recurrence, no significant differences were found in CD recurrence at 6–12 months follow-up, however KSA was ranked first based on SUCRA values, followed by EME, LME and other anastomosis. There were six direct comparisons between KSA and other anastomosis, four comparing EME to LME and one study comparing KSA to EME with 4 events of recurrence in the KSA group and 6 in the EME group allowing for improved indirect comparison compared to the indirect statistical analysis of the respective surgical recurrence [17].

The KSA gained momentum particularly after the SuPREMe-CD study, where 6 month endoscopic recurrence (Rutgeerts ≥ i2) was significantly reduced in the KSA group (22.2%) compared to STSA group (62.8%) with OR 5.91 (*p* < 0.001) and longer period to clinical recurrence [18]. A major limitation of this study as well as others included in this network meta-analysis is that they graded endoscopic recurrence based on the original Rutgeerts score of ≥ i2, where 5 or more ulcers were found either at the anastomosis or at the terminal ileum. Ulcers along an anastomotic staple line are not an uncommon finding and can be present without true CD recurrence. Thus, the score was later modified to account for this with separation into i2a (5 or more ulcers at the anastomosis), and i2b (5 or more ulcers at the terminal ileum), with the latter more predictive of endoscopic CD recurrence [19]. Only three of the studies in this study used Rutgeerts score ≥ i2b to define endoscopic recurrence. Two of these compared KSA to STSA (one RCT and one cohort study), and the other was an RCT comparing EME vs. LME. None of these studies found any significant difference in endoscopic recurrence [13, 20, 21] The lack of difference in endoscopic recurrence between the groups, despite there being reduction in surgical recurrence in KSA compared to other anastomosis may be explained by fewer studies using the modified Rutgeerts score, causing there to be an overrepresentation of endoscopic recurrence in other studies that used to tradition Rutgeerts score. Though it is also possible that surgical recurrence itself is influenced by heterogenous factors that play into the decision to operate or manage the patient medically, making it a difficult endpoint to replicate between studies.

Another limitation of this study is the construct of cohort studies where the other anastomosis or LME groups were often from a historic cohort that compared KSA or EME group. Over time, the development of biologics has changed the landscape of CD, inducing and maintaining improved remission. Five of the 10 cohort studies had higher rates of pre- or post-operative biological use in the KSA or EME group compared to other anastomosis or LME respectively [6, 8, 14, 22, 23]. This may have introduced temporal bias, where the biologics may have played a role in maintaining post-operative remission rather than the surgery itself. In each of these studies where surgical recurrence was assessed, it was significantly lower in the KSA or EME group compared to other anastomosis or LME respectively. Unfortunately, only one RCT comparing surgical recurrence in KSA, constituting level 1b evidence, the SuPREMe-CD study was present, drawing greater influence of results from cohort studies [18]. As such prospective cohort studies or RCTs with longer term follow-up would be useful in reducing the confounding effects from biologics.

Furthermore, the other anastomosis group included both STSA and end to end anastomosis (ETEA). This may have underrepresented the strength of the STSA as it has been found to be similar or superior in preventing CD recurrence compared to ETEA [24, 25].

Overall, we must take into consideration what type of recurrence actually matters clinically. The presence of endoscopic recurrence from ulcers at the anastomosis or terminal ileum may necessitate changes to patient medications particularly in the symptomatic individual, but it may not necessarily warrant intestinal resection. As such, surgical recurrence represents a definitive clinical outcome, whereas, as we have discussed, endoscopic recurrence is a surrogate marker but limited by variability and evolution in scoring systems. Thus, the lower surgical recurrence in KSA compared to other anastomosis may be a more compelling finding, despite no significant reduction in endoscopic recurrence in the same comparison of the two groups.

Our study is consistent with the ECCO guidelines that there is currently insufficient evidence to recommend EME over LME. Whilst our study finds that KSA is superior to other anastomosis with regards to reduction in surgical recurrence at 5 years, these findings must be interpreted with caution due to the aforementioned limitations. Despite this, the KSA was not been found to be inferior to other anastomotic configurations with respect to CD recurrence, which is also supported in the latest ECCO guidelines [5].

Future studies comparing KSA to stapled STSA, as well as EME and LME combined with either KSA and/or stapled STSA is required. This along with endoscopic recurrence being defined as Rutgeerts ≥ i2b and measuring surgical recurrence beyond 5 years is expected to help inform surgeons on the most optimal anastomotic technique in CD. The authors eagerly await the results of three active RCTs that are attempting to provide further evidence: the European HAND2END and End2End studies will look at KSA against other anastomosis (due to be completed in 2028), the international MESOCOLIC trial will compare LME and EME (due to be completed in 2025) and the four by four MEErKAT trial (UK) which will compare KSA and EME independently and as a combined approach (due to be completed in 2026).

## Conclusion

New surgical techniques have emerged over the last two decades for CD with the KSA establishing as an alternative anastomotic technique to the stapled STSA, and EME being investigated for its efficacy in reducing CD recurrence. Our network meta-analysis demonstrated a reduction surgical recurrence at 5 years with the KSA compared to other anastomosis but no significant differences endoscopic recurrence between KSA and other anastomosis or between EME and LME. Surgical recurrence between EME and KSA could not be appropriately compared due to limited number of studies. However, we advise surgeons to interpret the results within the limits of differing definitions of endoscopic recurrence, temporal bias from the effects of biological therapy, and the retrospective nature of some cohort studies. Further research directly comparing anastomotic and mesenteric techniques are required.

## Supplementary Information

Below is the link to the electronic supplementary material.

Supplementary Material 1

Supplementary Material 2

## Data Availability

The data that supports the findings of this study are available in the supplementary material of this article.

## Declarations

### Manuscript category

Review (network meta-analysis).
