# Supplementary material for: Preventing Crohn’s recurrence, Kono-S and extended mesenteric excision: network meta-analysis category
Source: Langenbecks Arch Surg. 2026 Jul 15;411(1):273. doi: 10.1007/s00423-026-04103-5 (PMC13658377; doi:10.1007/s00423-026-04103-5)
Supplement: Supplementary file 1 [file 423_2026_4103_MOESM1_ESM.docx]

**Preventing Crohn’s Recurrence, Kono-S and Extended Mesenteric Excision: Network Meta-Analysis**

Talia Shepherd^1^, Devansh Shah^1,2^, Joseph Do Woong Choi^1,3^, Toufic El-Khoury^1,3,4^, Nimalan Pathma-Nathan^1,3^, Amy Cao^1,3^, James Wei Tatt Toh^1,3^

1 – Department of Colorectal Surgery, Westmead Hospital, Westmead, New South Wales, Australia

2 – The University of Sydney, Nepean Hospital, Penrith, New South Wales, Australia

3 – The University of Sydney, Westmead Hospital, Westmead, New South Wales, Australia

4 – The School of Medicine, University of Notre Dame, New South Wales, Australia

**Corresponding Author**: James Wei Tatt Toh

- Postal Address: Westmead Hospital, Cnr Mons and Darcy Rd, Westmead, NSW, Australia, 2145
- Email: [james.toh@health.nsw.gov.au](mailto:james.toh@health.nsw.gov.au)
- ORCID ID: https://orcid.org/0000-0002-0110-2629

**Supplementary Tables**

| **Study** | **Country** | **Published evidence** | **Comparators** | **Design** | **Data collection time period** | **Population** | **Excluded** | **Intervention** | **Method of detecting recurrence** | **Follow-up** | **Primary Outcome** | **Secondary outcomes and important features** |
| --- | --- | --- | --- | --- | --- | --- | --- | --- | --- | --- | --- | --- |
| Kelm et al  2022^26^ | Germany | Full journal article | KSA vs STS (stapled) | Retrospective cohort study | 2019 - 2021 | 51 patients  Age 37 (16 - 71)  M:F 28:23  Mean BMI 23.45 | Extended resection or stricturoplasty, diverting stoma, hx of ulcerative colitis | Ileocolic resection.  KSA (n = 22) vs STS (n = 29).  STS was anisoperistaltic stapled. | Endoscopic (Rutgeerts score ≥i2) at 6-12 months | 5-15 months, median 8.8 months | No significant difference in ER (STS 13 vs KSA 7; p = 0.36) | Trend towards more severe inflammation in the STS group (mean Rutgeerts score 2.5 vs 1.7, p = 0.11). No difference in postoperative outcomes. |
| Shimada et al  2019^23^ | Japan | Full journal article | KSA vs ETEA | Retrospective cohort study | 2006 - 2016 | 215 patients  Age 37 (30 -45)  M:F 158:57  BMI 18.7 (16.9 - 20.2) | Low rectal anastomosis below the peritoneal fold | All bowel resections included (small bowel 117, large bowel 11 and mixed 86). KSA (n = 117) vs. handsewn ETEA (n = 98) | Surgical recurrence defined as a stricture or penetrating lesion at the anastomosis requiring surgical repair. Detected endoscopically, radiological, pathological (Crohn’s within resected specimens). | 10 years but overall median 54 months (IQR 29.7 - 86.3). Note that KSA had significantly shorter duration of follow-up due to study design (median 38 months vs 89 months in ETEA group, p<0.001) | SR significantly lower in KSA (4 vs 24, p<0.001) | 5-year surgery-free survival was significantly higher in KSA 95% (95% CI 0.9 - 1) vs ETEA 81.3% (95% CI 0.73 - 0.89). When data adjusted to 1 year, anastomotic configuration made no difference to SR, while anastomotic leakage was significantly associated (OR 23.6; 95% CI 3.9 - 14; p <0.001). Significantly higher rate of anastomotic leak in the ETEA group compared to KSA (6 vs 17, p=0.007) but all leaks were treated conservatively with drains (i.e. no reoperations). |
| Kono et al  2011^6^ | Japan | Full journal article | KSA vs other | Retrospective cohort study | 1993 - 2003 | 142 patients  Age 30 (14 - 62)  M:F 115:27 | Reoperation at another site (than initial anastomosis) | Small bowel and colonic resections included; KSA (n=69) vs other (n=73) (handsewn end to end or side to side or stapled side to side) with or without stricturoplasty | Endoscopic (Rutgeerts score ≥i2) at 6 months then annually by blinded endoscopists; surgical recurrence defined as reoperation for recurrent anastomotic disease | ER at 1 and 5 years, SR 5 years | No significant difference in ER at 1 year (KSA 49/59 vs other 58/73, p>0.05) or 5 years (KSA 69/69 vs other 73/73). Significantly lower SR in the KSA group at 5 years (0 vs 11; p = 0.0013). | Significantly higher Rutgeerts score in the "other" anastomosis group at 5 years (KSA 2.6 vs other 3.4; p= 0.008). |
| Coffey et al  2018^8^ | Ireland | Full journal article | EME vs LME | Retrospective cohort study | 2004 - 2010 (LME), 2010 - 2012 (EME) | 64 patients  Age 36 (24 - 51)  M:F 28:36 | Not available | Ileocolic resection. EME (n=34) vs LME (n=30). EME was defined as full mobilisation of the mesentery with division as close to the mesenteric root. | Surgical recurrence | EME mean 51.7 months ± 20.98, LME mean 69.9 months ± 48.47 | SR significantly lower in EME group (1 vs 9, p=0.003) | Multivariate analysis showing that LME is an independent risk factor for developing SR (p=0.007), as was smoking at time of surgery p=0.010 and disease phenotype p=0.048. |
| Mineccia et al (REMEDY)  2022^27^ | Italy | Full journal article | EME vs LME | Retrospective cohort study | 2009 - 2019 | 326 patients  Age 40.6 (24.6 - 56.6) | Jejuno-ileal joins, colonic resections | Primary ileocolic resection. EME (n=204) vs LME (n=122). One hospital (MH) divided mesentery flush with the bowel, while the second (FSH) performed ligation of the vessels at a D2 level to include all of the thickened mesentery and nodes. All anastomoses were STS (isoperistaltic for LME group and functional end to end for EME group). | Endoscopic (Rutgeerts score score ≥i2) at 6-12 months, ultrasonographic (bowel wall thickening >4mm at site of ileocolic anastomosis) at 6-12 months and surgical recurrence (needing a procedure at the site of anastomosis or any other intestinal segment) | 6-12 months ER, 5 years SR | No difference in ER (EME 91 vs LME 57, p=0.7) or USS recurrence (EME 40.4% vs 41.1%, p=0.9). No difference in SR (EME 2.8% vs 4%) at 5 years. | Significantly more stapled anastomosis and laparoscopic operations in EME group (more handsewn in LME group). No significant differences in postoperative outcomes. Found that postoperative therapy was the only thing that significantly reduced recurrence. Note: USS recurrence not able to be reported as a raw number of patients from the paper as only 290/326 pts in total had USS assessment and authors dont report how many in each group but only percentage. |
| Luglio et al (SUPREME-CD)  2020^18^ | Italy | Full journal article + abstract of the trial update (ECCO conference) | KSA vs STS (stapled) | single blind RCT (only patients blinded) | 2015 - 2017 | 119 patients  Age 38.9 (25-60) | Ostomy, pregnancy, contraindication to subsequent endoscopy | Ileocolic resection. KSA (n=51) vs STS (n=68). 1:1 randomisation. | Endoscopic (Rutgeerts score ≥i2) at 6 and 18 months; clinical (CDAI score >200) 3/6/9/12/15/18/24 months; surgical (need for reoperation in presence of symptoms) at 24 months | 5 years | Significantly lower ER at 6 months in KSA group (17 vs. 51, p<0.001). Significantly lower SR at 54 (42-60) months in KSA (0% vs 8.8%, p=0.02) | Significantly lower rate of severe ER (>i3) in KSA (8 vs 23, p=0.03). Significantly lower SR at 36 months in KSA (0 vs 6, p=0.03) but not at 24 months (0 vs 3, p=0.2). No significant difference in CR at 24 or 36 months (KSA 19.6% and 19.6% vs 30.9% and 33.8%, p=0.2 and 0.1 respectively). No differences in postoperative outcomes |
| Zhu et al  2021^28^ | China | Full journal article | EME vs LME | Retrospective cohort study | 2000 - 2018 | 126 patients  Age 30.77 (SD 10.21)  BMI 18.2 (SD 2.76) | Age <18, hx of colorectal resection, concomitant jejunal or ileal resection, APR, faecal diversion without resection, surgery for dysplasia or cancer complicating Crohn’s | Small bowel, colon and rectal operations included. EME (n=66) and LME (N=60). EME defined as resection within 1cm from origin of major arterial trunks. | Surgical recurrence | EME 47.5 months ± 23.67 and LME 45.12 months ± 25.45. Total about 75 months. | Significantly lower SR in EME group (7 vs 18, p=0.01). This differencestayed the same when subgroup analysis was done on only patients who had an anastomosis (this study did not exclude stomas) (EME 6 vs LME 14, p=0.01). | LME (and smoking) was an independent predictor for surgical recurrence in multivariate analysis. Kaplan meier curve showed EME had significantly longer post-op recurrence free survival (p=0.03) .Recurrences in EME group occurred at 31.57 ± 19.43 months, vs LME at 34.94 ± 22.89 months. Significantly higher blood loss in the LME group (p=0.002) but otherwise no difference in periop outcomes. |
| Alibert et al (KOCORICCO)  2023^22^ | France | Full journal article | KSA vs STS | Multicentre prospective, propensity matched cohort study- retrospective STS cohort vs prospective KSA | 2020 - 2022 (prospective) and 2013 - 2015 (retrospective) | 183 patients  Age 35 (27 - 50)  M:F 81:102  BMI 21.2 (19 - 24.9) | Emergency surgery, defunctioning stoma, lack of post-op clinical, biochemical or endoscopic data | Ileocolic resection. KSA (n = 61) vs STS (n = 122). STS included stapled or handsewn, any configuration. | Endoscopic (Rutgeerts score ≥i2) | 6-9 months | No difference in ER (KSA 29 vs STS 54, p=0.67) | No difference in severe ER (>i3) with KSA (7 vs 25, p=0.13). No difference in clinical recurrence but data only available for 106 pts (KSA 5 vs STS 12, p=1). KSA associated with significantly shorter LOS (6 days vs 6.5 days, p=0.001), lower chance of open or conversion to open (p=0.10) and significantly lower overall morbidity (9 vs 36, p=0.025). Note: I used the propensity matched data as raw groups were very unbalanced in terms of baseline characteristics and interpretation of that data would be difficult. Propensity matching was done on sex, smoking status, previous resection, penetrating behaviour, associated perianal disease, prior anti TNF therapy and postop biologic therapy. |
| Holubar et al  2024^5, 16, 17^ | USA | Full article and abstract P420 from ECCO conference 2023 | KSA vs EME vs KSA+EME | Retrospective cohort study | 2015 - 2022 | 186 patients  Age 37.5 (± 15.3) M:F 90:96  BMI 25.5 ± 5.8 | Not available | Ileocolic resection. KSA (n=74) vs EME (n=66) vs KSA+EME (n=46). Mesentery divided at the base of ileocolic pedicle for EME. | Endoscopic (Rutgeerts score ≥i2) and surgical (defined as disease progression requiring redo ileocolic resection) | Median 8 months (0-86). KSA 5 months (0-86); EME 18.5 months (0-48) and KSA+EME 7 months (0-25) | No difference in ER (KSA 4/20 vs EME 6/31 vs KSA+EME 3/18, p=0.69) or SR (KSA 1 vs EME 0 vs KSA+EME 1, p=0.22) | Significantly longer operating time (KSA 170 min vs EME 152 mins vs KSA+EME 187mins, p<0.01) but otherwise no differences in postop outcomes |
| Van Der Does De Willebois et al (SPICY trial)  2024^21^ | Netherlands and Italy | Full journal article | EME vs LME | RCT double blind (patient and endoscopic scoring), multicentre | 2020 - 2023 | 131 patients  Age 36 (IQR 25-54) | Emergency op, pregnancy, inability to comply with post op assessment | Ileocolic resection only. EME (n=66) vs LME (n=65). 139 enrolled but then 2 lost to f/up, 2 protocol deviations, 2 withdrew consent, 2 dx other than Crohn’s, 2 no anastomosis performed. | Endoscopic (Rutgeerts score ≥i2b) at 6 months | 6 months | No difference in ER (EME 28 vs LME 28, p=1) | Anastomotic leak rate higher in EME group (5/66 vs 1/65, p=0.208) but no difference in postop complications Clavien-Dindo grade IIIa or higher. |
| Trencheva et al  2024^13^ | International multicentre (USA, Germany, Italy) | Abstract OP20 from ECCO conference 2024 | KSA vs STS | RCT | Not available | 288 patients  Age 34 (18-81)  M:F 142:146  BMI 23.4 (SD 4.5) | Emergency resections, pregnancy, age < 18, recurrent or multisite Crohn’s, needing post op medical treatment for Crohn’s within 3-6 months | Ileocolic resection. KSA (n=154) vs STS (n=134) | Endoscopic (Rutgeerts score ≥i2b) | 30 days post op, scope at 3-6 months | No difference in ER at 3-6 months (KSA 35/135 vs. STS 32/115, p=0.883). | Trial not yet complete - baseline characteristics not evenly matched with significantly more smokers in KSA group (p=0.004). Only 81% patients have completed endoscopic surveillance at 3-6 months. No differences in surgical outcomes. |
| Tyrode et al  2024^20^ | France | Full journal article | KSA vs STS (all handsewn except for 1) | Combined cohort study - Kono S prospectively done, retrospective comparison cohort | 2020 - 2022 (retrospective 2010 - 2020) | 85 patients  Age 34.9 (27.6 - 45.6)  M:F 37:48  BMI 22.8 (IQR 19.9 - 26.2) | No endoscopic follow-up, post-op anastomotic fistula requiring revision, pre-emptive diverting stoma | Ileocolic resection. KSA (n=30) vs STS (n=55) | Endoscopic (Rutgeerts score ≥i2), surgical (new resection at anastomosis site at 1 year), clinical (Harvey Bradshaw index >4) and biochemical (faecal calprotectin > 100 at 6-12 months) | 6-12 months | No significant difference in ER using either Rutgeert's score (KSA 17 vs STS 27; p = 0.5) or modified Rutgeerts score (≥i2b) (KSA 14 vs STS 22, p=0.55). No surgical recurrence in either group at 12 months. | No difference in severe ER rate (>i3) in KSA (7 vs 10, p=0.57). No surgical recurrence in either group at 12 months. No difference in clinical recurrence (KSA 16 vs STS 28, p=0.18) or biological recurrence (KSA 4 vs STS 7, p=0.2). No significant differences in surgical outcomes or complications. |
| Uchino et al  2024^14^ | USA | Abstract P575 from ECCO conference 2024 | KSA vs STS (stapled) | Retrospective cohort study | 2020 - 2022 | 174 patients  BMI lower in the KSA group (24.3 vs. 26.4, p=0.04) | Other types of anastomosis | Ileocolic resection. KSA (n=83) vs STS (n=91) | Endoscopic (Rutgeerts score ≥i2) | 12 months | No difference in recurrence free survival between KSA and STS but multivariate analysis shows KSA associated with lower risk of perianastomotic recurrence requiring surgical/endoscopic intervention (OR 0.09, 95% CI 0.01-0.95, p=0.05). ER not significant (KSA 12 vs. STS 14; p=0.81). | Longer operative time in KSA group (186 vs 146 minutes, p=0.001), KSA more likely to have mesenteric excision although not defined in abstract (53% vs 15.4%, p=0.001), no significant differences in short term post-op outcomes. KSA group received more post-op biologics (65.1% vs 39.6%, p=0.001) and more frequently had medical therapy for perianastomotic recurrence (90% vs 68% p=0.14). |

***Table S1***: Full table – study characteristics of studies includes in network meta-analysis

| **Treatment Name** | **Kono-S** | | **Other anastomosis** | | **Limited mesenteric excision (LME)** | | **Extended mesenteric excision (EME)** | |
| --- | --- | --- | --- | --- | --- | --- | --- | --- |
| **Study Name** | **no. events** | **no. patients** | **no. events** | **no. patients** | **no. events** | **no. patients** | **no. events** | **no. patients** |
| **Tyrode *et al*** | 16 | 30 | 28 | 55 |  |  |  |  |
| **Luglio *et al*** | 3 | 36 | 8 | 43 |  |  |  |  |

***Table S2***: Summary of events in each study for clinical recurrence in 1 year

***Table S3***: Network Characteristics – assessing primary outcome of surgical recurrence at 5 years

***Table S4***: Intervention Characteristics – assessing primary outcome of surgical recurrence at 5 years

***Table S5***: Direct Comparison Characteristics – assessing primary outcome of surgical recurrence at 5 years

| **5 YEAR SURGICAL RECURRENCE** | | | | | | | | |
| --- | --- | --- | --- | --- | --- | --- | --- | --- |
| **Treatment Name** | **KSA** | | **Other anastomosis** | | **LME** | | **EME** | |
| **Study Name** | **no. events** | **no. patients** | **no. events** | **no. patients** | **no. events** | **no. patients** | **no. events** | **no. patients** |
| **Shimada *et al*** | 4 | 117 | 24 | 98 |  |  |  |  |
| **Kono *et al*** | 0 | 69 | 11 | 73 |  |  |  |  |
| **Luglio *et al* (SUPREME CD)** | 0 | 51 | 6 | 68 |  |  |  |  |
| **Holubar *et al*** | 1 | 74 |  |  |  |  | 0 | 66 |
| **Tyrode *et al*** | 0 | 30 | 0 | 55 |  |  |  |  |
| **Coffey *et al*** |  |  |  |  | 9 | 30 | 1 | 34 |
| **Minecchia *et al* (REMEDY)** |  |  |  |  | 4 | 122 | 9 | 204 |
| **Zhu *et al*** |  |  |  |  | 7 | 60 | 18 | 66 |

***Table S6***: Summary of events in each study for surgical recurrence at 5 years

| **Treatment** | **SUCRA** |
| --- | --- |
| **Limited mesenteric excision (LME)** | 0.8644 |
| **Extended mesenteric excision (EME)** | 0.8017 |
| **Kono-S** | 0.3338 |
| **Other anastomosis** | 0.741 x 10^-5^ |

***Table S7***: SUCRA probabilities for efficacy of KSA, other anastomosis, EME, and LME for reducing CD endoscopic recurrence at 6-12 months

***Table S8***: Network Characteristics – assessing primary outcome of endoscopic recurrence at 6-12 months

***Table S9***: Intervention Characteristics – assessing primary outcome of endoscopic recurrence at 6-12 months

***Table S10***: Direct Comparison Characteristics – assessing primary outcome of endoscopic recurrence at 6-12 months

| **Treatment Name** | **KSA** | | **Other anastomosis** | | **LME** | | **EME** | |
| --- | --- | --- | --- | --- | --- | --- | --- | --- |
| **Study Name** | **no. events** | **no. patients** | **no. events** | **no. patients** | **no. events** | **no. patients** | **no. events** | **no. patients** |
| **Kelm *et al*** | 7 | 22 | 13 | 29 |  |  |  |  |
| **Kono *et al*** | 49 | 59 | 58 | 73 |  |  |  |  |
| **Luglio *et al* (SUPREME CD)** | 17 | 51 | 51 | 68 |  |  |  |  |
| **Alibert *et al* (KOCORICCO)** | 29 | 61 | 54 | 122 |  |  |  |  |
| **Trencheva *et al*** | 35 | 135 | 32 | 115 |  |  |  |  |
| **Tyrode *et al*** | 17 | 30 | 27 | 55 |  |  |  |  |
| **Uchino *et al*** | 12 | 83 | 14 | 91 |  |  |  |  |
| **Holubar *et al*** | 4 | 20 |  |  |  |  | 6 | 31 |
| **Minecchia *et al* (REMEDY)** |  |  |  |  | 57 | 122 | 91 | 204 |
| **Van der Does de Willebois *et al* (SPICY)** |  |  |  |  | 28 | 65 | 28 | 66 |

***Table S11***: Summary of events in each study for endoscopic recurrence at 6-12 months

| **Kono-S** |  |  |  |
| --- | --- | --- | --- |
| **1.06 (0.20 – 5.14)** | **Extended mesenteric excision (EME)** |  |  |
| *0.99 (0.17 – 5.42)* | **0.94 (0.53 – 1.68)** | **Limited mesenteric excision (LME)** |  |
| **0.78 (0.54 – 1.13)** | *0.74 (0.14 – 4.02)* | *0.79 (0.14 – 4.72)* | **Other anastomosis** |

***Table S12***: Pairwise comparison table – efficacy of KSA, other anastomosis, EME, and LME for reducing CD endoscopic recurrence at 6-12 months in network meta-analysis reported as odds ratio (95% credible interval). Direct comparisons are represented in bold, and indirect comparisons are represented in italics.

**Supplementary Figures**

***Figure S1***: Rankogram with probability for ranking 1st to 4th (best to worst respectively) with regards to efficacy of KSA, other anastomosis, EME, and LME for reduction in CD endscopic recurrence, ranging from 1.0 (most likely to fit in assigned ranking) to 0.0 (least likely to fit in assigned ranking) on the Y-axis. Ranking of interventions displayed on the X-axis ranging from 1st to 4th.

***Figure S2***: Forest Plot comparing interventions (KSA, other anastomosis, EME, and LME) assessed with relation to CD endoscopic recurrence. Line of no effect at OR = 1.0
